# Metabolic Variations of Flavonoids in Leaves of *T. media* and *T. mairei* Obtained by UPLC-ESI-MS/MS

**DOI:** 10.3390/molecules24183323

**Published:** 2019-09-12

**Authors:** Tao Wang, Fengjiao Zhang, Weibing Zhuang, Xiaochun Shu, Zhong Wang

**Affiliations:** Jiangsu Key Laboratory for the Research and Utilization of Plant Resources, Institute of Botany, Jiangsu Province and Chinese Academy of Sciences (Nanjing Botanical Garden Mem. Sun Yat-Sen), Nanjing 210014, Jiangsu, China; johnwt1007@163.com (T.W.); fengjiao@cnbg.net (F.Z.); wanmeiabc@hotmail.com (W.Z.); islbe@163.com (X.S.)

**Keywords:** *Taxus media*, *Taxus mairei*, flavonoid metabolites, OPLS-DA, UPLC-ESI-MS/MS

## Abstract

The needles of *Taxus* species contain a large number of bioactive compounds, such as flavonoids. In the present study, the total flavonoid content in leaves of *Taxus media* and *Taxus mairei* was 19.953 and 14.464 mg/g, respectively. A total of 197 flavonoid metabolites (70 flavones, 42 flavonols, 26 flavone C-glycosides, 20 flavanones, 15 anthocyanins, 13 isoflavones, 6 flavonolignans, and 5 proanthocyanidins) were identified for the first time by a widely targeted Ultra Performance Liquid Chromatography-Electrospray Ionization-Tandem Mass Spectrometry (UPLC-ESI-MS/MS) method within the two *Taxus* species, containing 160 common metabolites, with 37 unique metabolites merely determined in *T. mairei* or *T. media*. Moreover, 42 differential flavonoid metabolites were screened in the two *Taxus* species, which showed specific metabolic patterns in isoflavonoid biosynthesis, anthocyanin biosynthesis, and flavone and flavonol biosynthesis pathways. Compared to *T. mairei*, a more activated phenylpropanoid pathway was found in *T. media*, which could be responsible for the higher content of total flavonoids in *T. media*. Our results provide new insights into the diversity of flavonoid metabolites between *T. mairei* and *T. media*, and provide a theoretical basis for the sufficient utilization of *Taxus* species and the development of novel drugs.

## 1. Introduction

Flavonoids are widely distributed phenolic compounds in the plant kingdom and occur in all parts of plants as complex mixtures of different components, including anthocyanins, flavanes, flavones, flavanones, flavonols, and chalcones [1,2]. They have many functions in plants, such as providing pigmentation to flowers, fruits, and seeds to attract pollinators and seed dispersers [3]; participating in defense and resistance responses during plant growth and development [4]; and improving the utilization of nutrient elements and the germination of pollen [5]. Moreover, they also display wide pharmacological activities, including anti-oxidative, anti-tumor, anti-leukemic, anti-inflammatory, and immunomodulatory effects [6,7,8].

*Taxus* species, also known as yew, represent a group of evergreen coniferous trees or shrubs, which belong to the genus *Taxus* and family *Taxaceae* [9]. Plants of the genus *Taxus* have attracted much attention around the world because of taxol, which is a diterpenoid substance originally isolated from the bark of *Taxus brevifolia* [10] and considered one of the most effective antineoplastic drugs for the treatment of breast, lung, and ovarian cancers [11]. To date, yew leaves are considered the most ideal and reliable source for the industrial extraction of taxol, because of their renewability [12]. Except for taxol, there are also many kinds of bioactive substances in leaves of *Taxus* species, such as flavonoids. Recently, many flavonols and biflavones were isolated from *Taxus* species, such as quercetin, ginkgetin, and amentoflavone [8,13]. These flavonoids have been proved to improve the anti-cancer efficacy and reduce the toxic and side effects in combination with taxol, providing a new approach for the cure of cancer and other diseases [14,15]. However, the flavonoids have not been extensively explored in *Taxus* species as major bioactive components, even though they are involved in many biological functions and have important health-related roles. There are still a large number of flavonoids that are expected to be effectively developed and utilized, especially from *Taxus* remainder extracts free of toxoids, which were previously derived from the branches and leaves of *Taxus* species for the industrial extraction of taxol [16].

*Taxus media* and *Taxus mairei* are listed as rare and endangered species [17,18], and are the precious and major source for industrial taxol production [9,19]. In the present study, leaves of *T. media* and *T. mairei* were investigated for the first time by a widely targeted Ultra Performance Liquid Chromatography-Electrospray Ionization-Tandem Mass Spectrometry (UPLC-ESI-MS/MS) method to reveal the various flavonoid metabolites and metabolic variations within two *Taxus* species. Our results will enrich the diversity of the flavonoid compounds in yew leaves, and provide a theoretical basis for utilizing *Taxus* species’ leaves and developing novel drugs.

## 2. Results

### 2.1. Determination of Total Flavonoid Content

**Figure 1:**
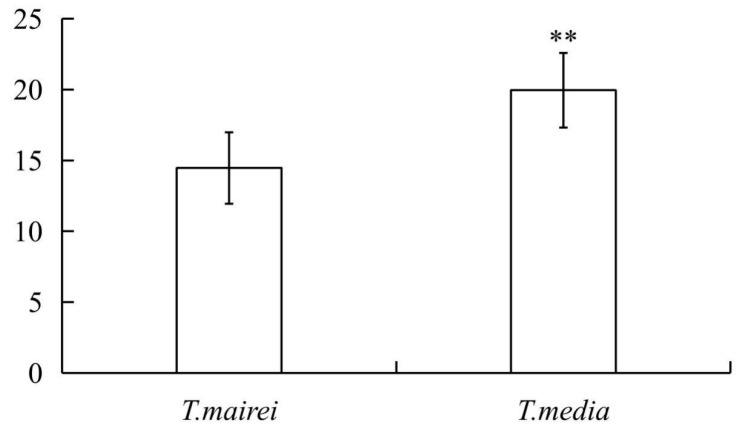
Variations of the total flavonoid contents in the needles of *Taxus mairei* and *Taxus media* samples. Significant variations (*p* < 0.01) are indicated by **. Error bars represent means ± SD (*n* = 8).

The total flavonoid contents in the leaves of *T. mairei* and *T. media* were determined and are presented in Figure 1. Among them, the flavonoid content in *T. mairei* was 14.464 mg/g DW, while that in *T. media* was 19.953 mg/g DW, and this was statistically significant (*p* < 0.01).

### 2.2. Metabolic Profiling of Two Taxus Species

**Figure 2:**
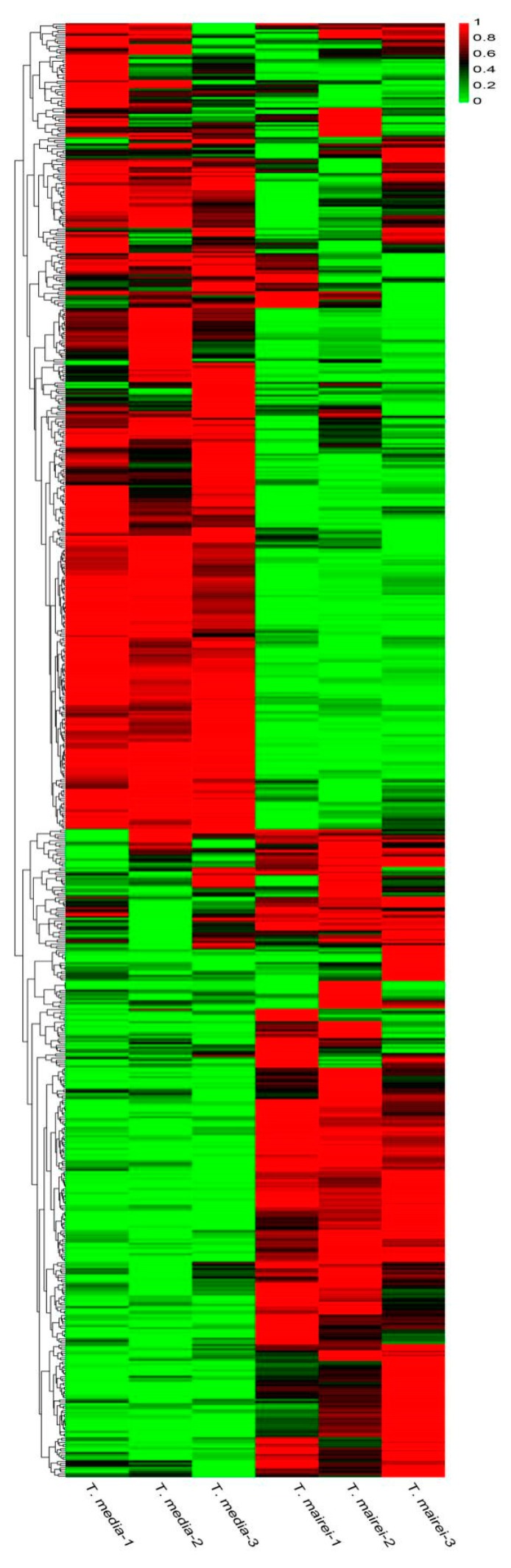
A heatmap of the metabolites identified in the metabolomes of the two *Taxus* species (*n* = 3). The content of each metabolite is represented by a color bar. With the increase in the metabolite content, the color of the bar changed from green to red.

A total of 689 annotated metabolites were identified in the leaves of *T. mairei* and *T. media* based on UPLC-ESI-MS/MS and databases. To examine the quality of the acquired MS data, total ion chromatograms (TICs) were generated for the samples and revealed a high degree of overlap (Supplementary Figure S1a,b). An overview of the metabolite profiling for *T. mairei* and *T. media* is shown in Figure 2 as a heat map, which exhibits dramatic variations in the metabolite contents. Details of the qualitative and quantitative analysis of metabolites can be seen in Supplementary Table S1.

**Figure 3:**
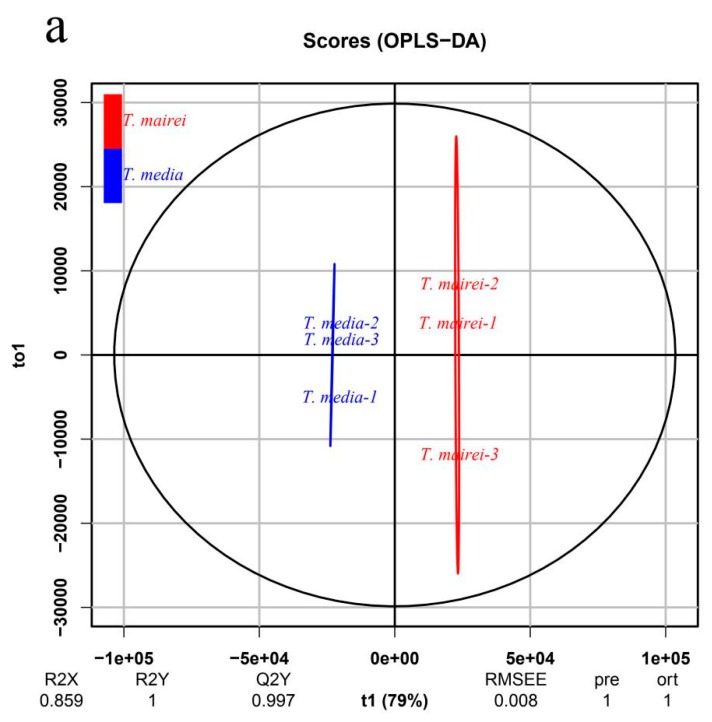

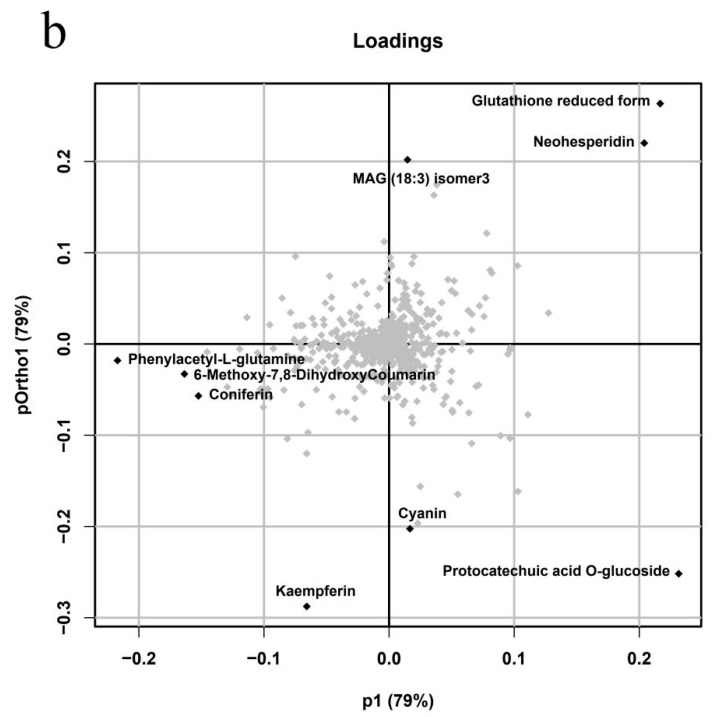
Differential metabolite analysis conducted by orthogonal projection to latent structures-discriminant analysis (OPLS-DA). (**a**) OPLS-DA score plot comparing *T. mairei* and *T. media*. (**b**) OPLS-DA loading plot comparing *T. mairei* and *T. media*.

**Figure 4:**
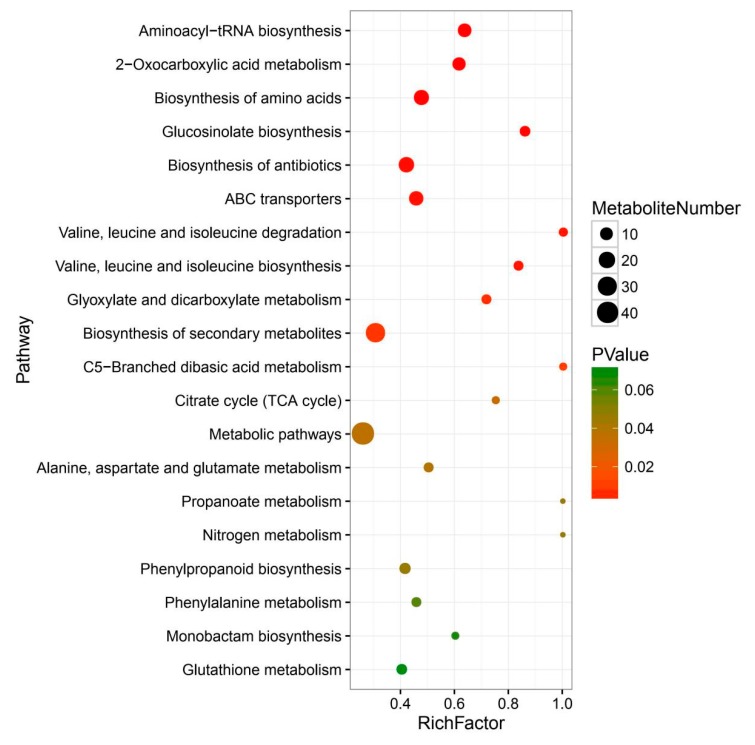
Kyoto Encyclopedia of Genes and Genomes (KEGG) pathway enrichments of differential metabolites in TOP20.

Moreover, the orthogonal projection to latent structures-discriminant analysis (OPLS-DA) model was constructed to evaluate the significance of the metabolic difference within two *Taxus* species. The score plot showed a clear separation between the *T. mairei* and *T. media*, indicating highly significant differences in the metabolite profiles of the two *Taxus* species (Figure 3a). Q^2^ was 97.1%, further indicating that the prediction ability of this model was excellent. The loading plot displayed the metabolites contributing to the differences between *T. mairei* and *T. media*, including neohesperidin, cyanin, and kaempferin (Figure 3b). According to the P-value of the T-test combined with variable importance in projection (VIP) values of the OPLS-DA model, 127 differential metabolites were screened and are shown in Supplementary Table S2. Based on their annotations given by the Kyoto Encyclopedia of Genes and Genomes (KEGG), the top 20 largest metabolic categories, including the biosynthesis of secondary metabolites (31 metabolites), biosynthesis of antibiotics (17 metabolites), and biosynthesis of amino acids (16 metabolites), are shown in Figure 4.

### 2.3. Metabolomics Analysis Reveals Variations in the Flavonoids

**Figure 5:**
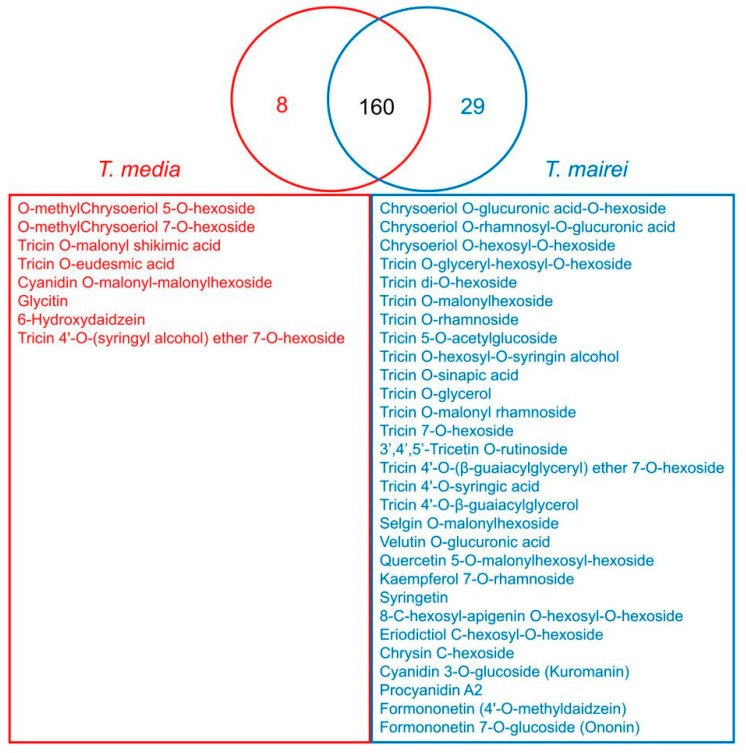
Venn diagram revealing the common and unique metabolites between *T. mairei* and *T. media*.

**Figure 6:**
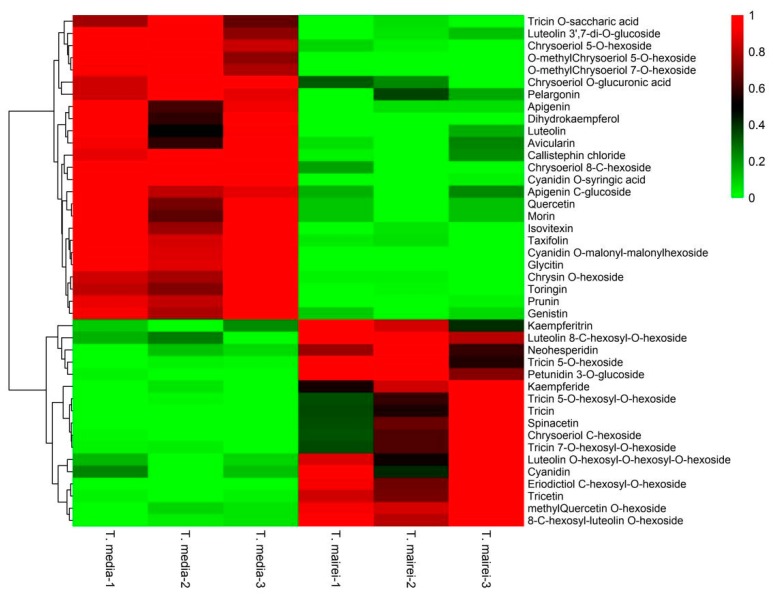
A heatmap of differential flavonoid metabolites between leaves of *T. mairei* and *T. media* (*n* = 3). The content of each differential flavonoid metabolite is represented by a color bar. With the increase in the metabolite content, the color of the bar changed from green to red.

In this study, 197 flavonoid metabolites were identified, including 70 flavones, 42 flavonols, 26 flavone C-glycosides, 20 flavanones, 15 anthocyanins, 13 isoflavones, 6 flavonolignans, and 5 proanthocyanidins (Supplementary Table S3). Among them, 160 flavonoid metabolites were common within the two *Taxus* species, while 8 were only found in *T. media*, and 29 were merely determined in *T. mairei* (Figure 5). According to the screening criteria of differential metabolites, 42 differential flavonoid metabolites were screened in the present study. Details of all differential flavonoid metabolites of the samples are shown in Figure 6 as a heat map. The contents of these metabolites in the two *Taxus* species were obviously distinguished, including 25 predominantly accumulated in *T. media*, and 17 significantly accumulated in *T. mairei*. The KEGG classification results indicated that the differential flavonoid metabolites of the two *Taxus* species were mainly involved in anthocyanin biosynthesis, flavone and flavonol biosynthesis, and isoflavonoid biosynthesis.

### 2.4. Metabolomics Analysis Reveals Variations in the Phenylpropanoid Pathway

The phenylpropanoid pathway is an important upstream pathway regulating the synthesis of flavonoid metabolites. Our results showed that phenylalanine, p-coumaric acid, caffic acid, and ferulic acid, which are the key metabolites in phenylpropanoid biosynthesis, were significantly accumulated in *T. media* rather than in *T. mairei*. Moreover, some derivatives of intermediates, such as caffeic acid O-glucoside, 3-O-p-coumaroyl shikimic acid O-hexoside, 3-O-p-coumaroyl quinic acid, and 3-hydroxy-4-methoxycinnamic acid, were also predominantly accumulated in *T. media*. Besides flavonoid metabolites, a high number of coumarins and their derivatives were observed to have a higher content in *T. media* than in *T. mairei*, which indicated a highly activated phenylpropanoid pathway in the leaves of *T. media*.

## 3. Discussion

Flavonoids comprise a large and diverse group of polyphenolic plant secondary metabolites, which has a broad application in drug development and health care [20,21]. In the present study, the total flavonoid content in leaves of *T. media* and *T. mairei* was 19.953 and 14.464 mg/g, respectively, which was similar to that in *Ginkgo bilabo* (14.8–24.9 mg/g), an important source for the extraction of flavonoids [22], indicating huge potentials for developing and utilizing flavonoids in yew leaves. Widely targeted metabolomics was used for the first time to investigate the diversity of flavonoid metabolites in the needles of *T. media* and *T. mairei*. A total of 197 flavonoid metabolites were identified, far exceeding the previously published metabolomes, and most of which were first reported in *Taxus* species. Through clustering analysis and OPLS-DA analysis, considerable differences of flavonoid metabolites were observed between *T. media* and *T. mairei*, and a series of unique flavonoid metabolites were also detected in the two *Taxus* species, respectively. Interestingly, *T. mairei* displayed more unique flavonoid metabolites than *T. media*, while the total flavonoid content in *T. media* was significantly higher than that in *T. mairei*. These results indicated different values for developing and utilizing flavonoids in the leaves of *T. media* and *T. mairei*. Moreover, compared to other studies, our results also uncovered the broadest metabolomics of *T. media* and *T. mairei*, containing 689 metabolites and the major metabolic pathways covered by 306 named metabolites from the KEGG database, which will be helpful for identifying the known and new metabolites and the possible relationship between the metabolites within two *Taxus* species, through the comprehensive analysis of the above metabolite expression profiles.

**Figure 7:**
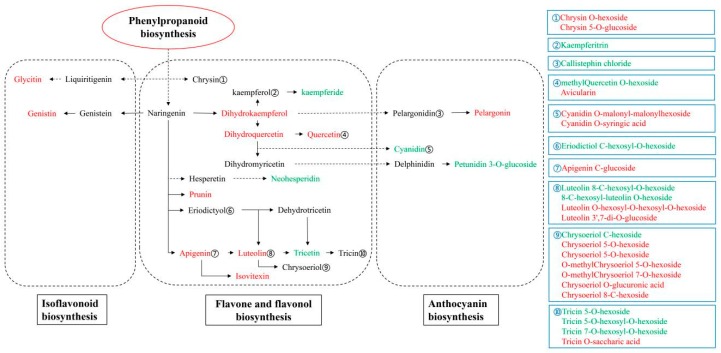
The differential flavonoid metabolites in the two *Taxus* species involved in anthocyanin biosynthesis, flavone and flavonol biosynthesis, and isoflavonoid biosynthesis. Red letters indicate the *T. media* predominantly accumulated metabolites; green letters indicate the *T. mairei* predominantly accumulated metabolites. Unannotated derivatives of flavonoid metabolites by KEGG are listed in the blue box.

To illustrate the metabolic variations of flavonoids in leaves between *T. media* and *T. mairei*, a schematic flow chart of the flavonoid pathway in the two *Taxus* species is shown in Figure 7, which mainly covers the differential flavonoid metabolites enriched in the flavone and flavonol biosynthesis, anthocyanin biosynthesis, and isoflavonoid biosynthesis pathways. Among them, two differential flavonoid metabolites, glycitin and genistin (genistein 7-O-glucoside), were identified in the isoflavonoid biosynthesis pathway. As the isomeric forms of flavones, isoflavones are recognized as weak phytoestrogens, which possess positive effects on anti-cancer, cardiovascular disease treatment, and the prevention and treatment of Alzheimer′s disease [23,24]. Tokalov et al. reported the protection of genistin on p53 wild-type cells from taxol in the combined treatment of lung cancer [25]. The protective effect of glycitin on UV-induced skin photoaging in human primary dermal fibroblasts and lipopolysaccharide-induced acute lung injury has been proved by Seo et al. and Chen et al., respectively [26,27]. In the present study, genistin was predominantly accumulated in *T. media* rather than in *T. mairei*, and glycitin was unique in *T. media*. These combined results indicated the specific medicinal potential of *T. media* leaves.

Compared to the isoflavonoid biosynthesis pathway, the flavone and flavonol biosynthesis pathway enriched the most differential flavonoid metabolites, including quercetin, prunin, apigenin, luteolin, and isovitexin predominantly accumulated in *T. media*, and kaempferide, neohesperidin, and tricetin predominantly accumulated in *T. mairei*. Moreover, a series of derivatives of the flavones and flavonols above also displayed differential accumulations in the two *Taxus* species. According to the results of peak area calculations, prunin and apigenin displayed higher accumulations in *T. media* than in *T. mairei*, indicating a strong reactive oxygen species (ROS) scavenging activity, and a medicinal potential against UV-induced skin tumorigenesis in *T. media* leaves [28,29]. The high level of neohesperidin in *T. mairei* leaves implied specific antioxidant, anti-inflammatory, and anti-allergic activities for preventing mast cell-immediate and delayed allergic diseases [30]. Quercetin is one of the most abundant dietary flavonoids [31]. Numerous in vitro and animal model studies using quercetin alone or quercetin in combination with other bioactive compounds have shown the anticancer activities of the compound [32,33,34]. To our surprise, Hao et al. reported that higher levels of the total and the individual flavonoid quercetin were detected in *T. mairei* than in *T. media* [35], which was opposite to our results. The flavonoid contents were greatly affected by environmental factors, such as temperature, and light [8,22]. Therefore, reasons for this variation in results could be the different cultivation conditions and growth environments of *Taxus* trees used in the experiments. Moreover, a series of derivatives of chrysoeriol and tricin, most of which were attached to glycosides, were unique in *T. mairei* or *T. media*. Numerous glycosides of chrysoeriol or tricin showed differential accumulations in the two *Taxus* species, while no significant difference was observed in the content of tricin and chrysoeriol. These results might imply specific glycoside-based regulatory modes in the two *Taxus* species. Tricin is a methylated flavone that has considerable potential as a functional agent for glycemic control and also has anti-inflammatory and anti-cancer effects [1]. Chrysoeriol not only possesses many biological effects similar to luteolin, such as antioxidant and anti-inflammatory effects, but also exhibits its own effects, such as osteoporosis [36]. The glycosides conferred a huge diversity to the structure of tricin and chrysoeriol, which may further enrich their biological functions and pharmacological effects [37].

It was noteworthy that no significant difference was detected in the content of pelargonidin or delphinidin enriched in the anthocyanin biosynthesis pathway, while their derivatives, namely pelargonin (pelargonidin 3, 5-diglucoside), callistephin chloride (pelargonidin 3-O-beta-D-glucoside), and petunidin 3-O-glucoside, were differentially accumulated in the two *Taxus* species. Moreover, the cyanidin was predominantly accumulated in *T. mairei*, while the cyanidin-based anthocyanins, namely cyanidin O-syringic acid and cyanidin O-malonyl-malonylhexoside, were predominantly accumulated in *T. media*. The cyanidin, pelargonidin, and delphinidin, and their derivatives, are considered the main components of anthocyanins, which confer a red, magenta, or blue hue to plants, respectively [1]. However, there was no significant difference in leaf color between the two *Taxus* species. Therefore, the differential cyanidin-, pelargonidin-, and delphinidin-based anthocyanins, most of which were first detected in *Taxus* species, might offer added biological functions due to their attached glycosides, in addition to the colors and health benefits already attributed to anthocyanin compounds [38,39,40]. Meanwhile, these flavonoid glycosides were also considered the most vital phytochemicals, which displayed an improved water solubility, stability, selectivity, and efficacy in the development of novel drugs [41,42].

**Figure 8:**
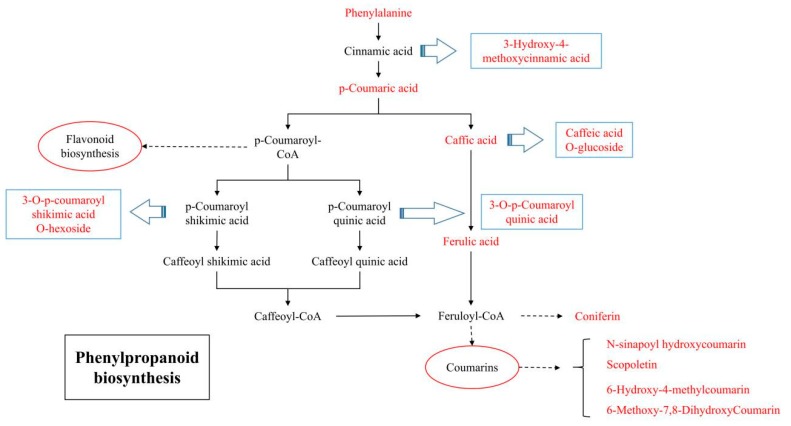
The differential flavonoid metabolites in the two *Taxus* species involved in the phenylpropanoid biosynthesis pathway. Red letters indicate the *T. media* predominantly accumulated metabolites; green letters indicate the *T. mairei* predominantly accumulated metabolites. Unannotated derivatives of flavonoid metabolites by KEGG are listed in the blue box.

Moreover, a series of key metabolites and their derivatives enriched in the phenylpropanoid pathway were highly accumulated in *T. media* leaves (Figure 8). The phenylpropanoid pathway is a ubiquitous and well-described plant secondary metabolite pathway; flavonoids, such as flavones, flavonols, and anthocyanins, are synthesized through this pathway [43]. Therefore, one of the main factors contributing to the higher content of total flavonoids in *T. media* was related to the activation of the phenylpropanoid pathway. In addition to flavonoids, the downstream products of the phenylpropanoid pathway included lignins, coumarins, and stilbenes [44]. In our study, a large number of coumarins and their derivatives were also predominantly accumulated in *T. media*, suggesting a stronger environmental adaptiveness in *T. media* than in *T. mairei* [45,46], which is helpful for the accumulation of flavonoids in different climates. Further quantitative analysis and separation of these flavonoid metabolites, and exploration of variations in the expression and activity of key enzymes catalyzing the synthesis of flavonoids and phenylpropanoids, could improve the comprehensive understanding of metabolic patterns of flavonoids in leaves of *Taxus* species [47].

## 4. Materials and Methods

### 4.1. Plant Materials

The individuals of *T. mairei* and *T. media* selected for this experiment were cultivated under the same maintenance conditions in a germplasm nursery for *Taxus* species. The germplasm nursery is located in Wuxi, Jiangsu Province, China (120°32′E, 31°43′N), which belongs to the humid subtropical climate zone. The annual average temperature is about 16.2 °C, and the average annual precipitation exceeds 1000 mm. Moreover, the soil of the site is sandy loam and is neutral (pH = 6.68). These climatic and environmental conditions are suitable for the growth and development of *Taxus* species. To maintain identical conditions, a sprinkler irrigation system was used to meet the water requirement of *Taxus* trees. They were sprayed once every 4–5 days and foliar spray was added in the early morning and evening in summer. In June 2017, fresh needles of triplicate samples were collected from 10 independent 12-year-old *Taxus* trees of *T. mairei* and *T. media*, respectively. The sampling site and time were the same. Samples were frozen in liquid nitrogen immediately after collection and then stored in −80 °C conditions.

### 4.2. Determination of Total Flavonoids Content

Needles collected from the two *Taxus* species were dried at 65 °C for about 6 h and powdered. The total flavonoid content of 0.1 g of leaves was determined in accordance with the protocol of the Plant Flavonoids Test kit (Solarbio Science & Technology Co., Ltd., Beijing, China).

### 4.3. Sample Preparation and Extraction

The freeze-dried samples were ground into powder and extracted overnight at 4 °C with 70% aqueous methanol containing 0.1 mg/L lidocaine for the internal standard. Then, the dissolved sample was centrifuged at 10,000× *g* for 10 min, and the supernatant was absorbed and filtrated (SCAA-104, 0.22 μm pore size; ANPEL, Shanghai, China) before UPLC-ESI-MS/MS analysis. Moreover, one quality control (QC) sample was inserted for every 10 test samples to detect the reproducibility of the whole experiment.

### 4.4. UPLC-ESI-MS/MS Analysis

The sample extracts were analyzed using a UPLC-ESI-MS/MS system (UPLC, Shim-pack UFLC SHIMADZU CBM20A; MS/MS, Applied Biosystems 4500 QTRAP). In total, 5 µL of samples was injected into a Waters ACQUITY UPLC HSS T3 C18 column (2.1 mm × 100 mm, 1.8 µm) operating at 40 °C and a flow rate of 0.4 mL/min. The mobile phases used consisted of phase A (0.04% acetic acid in water) and phase B (0.04% acetic acid in acetonitrile). The gradient elution procedures were set as follows: 95:5 Phase A/Phase B at 0 min; 5:95 Phase A/Phase B at 11.0 min; 5:95 Phase A/Phase B at 12.0 min; 95:5 Phase A/Phase B at 12.1 min; 95:5 Phase A/Phase B at 15.0 min. After UPLC, the effluent was alternatively connected to an ESI-triple quadrupole-linear ion trap (Q TRAP)-MS.

Linear ion trap (LIT) and triple quadrupole (QQQ) scans were acquired on a triple quadrupole-linear ion trap mass spectrometer (Q TRAP), AB Sciex QTRAP4500 System, equipped with an ESI-Turbo Ion-Spray interface, operating in both positive and negative ion mode and controlled by Analyst 1.6.1 software (AB Sciex). The operation parameters were as follows: electrospray ionization (ESI) source temperature was set at 550 °C; ion spray voltage (IS) was set at 5500 V; curtain gas (CUR) was set at 25psi; the collision-activated dissociation (CAD) was set at the highest value. QQQ scans were acquired as multiple reaction monitoring (MRM) experiments with an optimized de-clustering potential (DP) and collision energy (CE) for each individual MRM transition. The m/z range was set between 50 and 1000.

### 4.5. Data Pre-Processing and Metabolite Identification

The acquired MS data pretreatments, including data filtering, peak detection, alignment, and calculations, were performed using Analyst 1.6.1 software. Metabolites were identified by searching the internal database and public databases (MassBank, KNApSAcK, HMDB, MoTo DB, and METLIN) and comparing the *m*/*z* values, the retention time (RT), and the fragmentation patterns with the standards. To facilitate the identification of metabolites, an accurate m/z for each Q1 was obtained. Total ion chromatograms (TICs) of QC samples were exported to give an overview of the metabolite profiles of all samples. Then, the area of each chromatographic peak was calculated and peaks were aligned across the different samples based on the spectral pattern and RT. The analysis removed the redundant signals caused by different isotopes; in-source fragmentation; K^+^, Na^+^, and NH_4_^+^ adducts; and dimerization.

### 4.6. Statistical Analysis

The pre-processed dataset was fed into the R package model ropls (http://bioconductor.org/packages/release/bioc/html/ropls.html) for hierarchical clustering analysis (HCA), and orthogonal projection to latent structures-discriminant analysis (OPLS-DA). The OPLS-DA model was further validated by cross-validation. Moreover, the variable importance in projection (VIP) score of the OPLS model combined with the T-test was applied for screening differential metabolites. Those with a P-value of the T-test < 0.05 and VIP ≥ 1 were considered differential metabolites between the two *Taxus* trees of *T. mairei* and *T. media*. Subsequently, metabolites were mapped to Kyoto Encyclopedia of Genes and Genomes (KEGG) metabolic pathways for pathway analysis and enrichment analysis (http://www.genome.jp/kegg/). The figures used in this article were drawn by using Microsoft Office Excel 2011 coupled with Adobe Illustrator CC.

## 5. Conclusions

The needles of *T. media* and *T. mairei* are rich in flavonoids. The total flavonoid content in leaves of *T. media* (19.953 mg/g) was significantly higher than that of *T. mairei* (14.464 mg/g). Widely targeted UPLC-ESI-MS/MS metabolomics was conducted for the first time to investigate various flavonoid metabolites and metabolic variations in leaves of the two *Taxus* species. This represents the broadest metabolomics study of *T. media* and *T. mairei* so far, and 689 metabolites and the major metabolic pathways covered by 306 named metabolites from the KEGG database were shown in the present study. Among them, a total of 197 flavonoid metabolites were identified, including 70 flavones, 42 flavonols, 26 flavone C-glycosides, 20 flavanones, 15 anthocyanins, 13 isoflavones, 6 flavonolignans, and 5 proanthocyanidins, most of which were reported for the first time in this study for *Taxus* species. Moreover, both *Taxus* species displayed unique flavonoid metabolites, indicating specific medicinal potential for developing and utilizing flavonoids in *Taxus* species. According to the P-value of the T-test combined with the VIP values of the OPLS-DA model, 42 differential flavonoid metabolites were screened and were mainly enriched in isoflavonoid biosynthesis, anthocyanin biosynthesis, and flavone and flavonol biosynthesis pathways, which showed specific metabolic patterns between *T. mairei* and *T. media*. Compared with *T. mairei*, a more activated phenylpropanoid pathway was found in *T. media*, which was considered an important factor contributing to the higher flavonoid content accumulated in *T. media*. Further comprehensive analysis of the expression profiles of these metabolites and the possible relationship could provide a basis for the development and utilization of flavonoid metabolites in *Taxus* species.

## Supplementary Materials

Supplementary Materials are available online. Figure S1: The stacking diagram of total ions current (TIC) maps from quality control samples (QC) mass spectrometry. (a) TIC of positive ion multiple reaction monitoring (MRM). (b) TIC of negative ion MRM; Table S1: Qualitative and quantitative analysis of metabolites by UPLC-ESI-MS/MS in the two *Taxus* species; Table S2: Qualitative and quantitative analysis of the 127 differential metabolites detected in the two *Taxus* species; Table S3: Qualitative and quantitative analysis of the 197 flavonoid metabolites detected in the two *Taxus* species.
