# Supplementary material for: Metabolic Variations of Flavonoids in Leaves of T. media and T. mairei Obtained by UPLC-ESI-MS/MS
Source: Molecules. 2019 Sep 12;24(18):3323. doi: 10.3390/molecules24183323 (PMC6767174; doi:10.3390/molecules24183323)
Supplement: Supplementary file 1 [file molecules-24-03323-s001.zip › supplementary files/Fig legends.docx]

Fig legends

Fig. S1 The stacking diagram of total ions current (TIC) maps from quality control samples (QC) mass spectrometry. (a) TIC of positive ion multiple reaction monitoring (MRM). (b) TIC of negative ion MRM.

Table S1 Qualitative and quantitative analysis of metabolites by UPLC-ESI-MS/MS in the two *Taxus* species.

Table S2 Qualitative and quantitative analysis of the 127 differential metabolites detected in the two *Taxus* species.

Table S3 Qualitative and quantitative analysis of the 197 flavonoid metabolites detected in the two *Taxus* species.
