# Supplementary figures and images for: Metabolic Variations of Flavonoids in Leaves of T. media and T. mairei Obtained by UPLC-ESI-MS/MS
Source: Molecules. 2019 Sep 12;24(18):3323. doi: 10.3390/molecules24183323 (PMC6767174; doi:10.3390/molecules24183323)

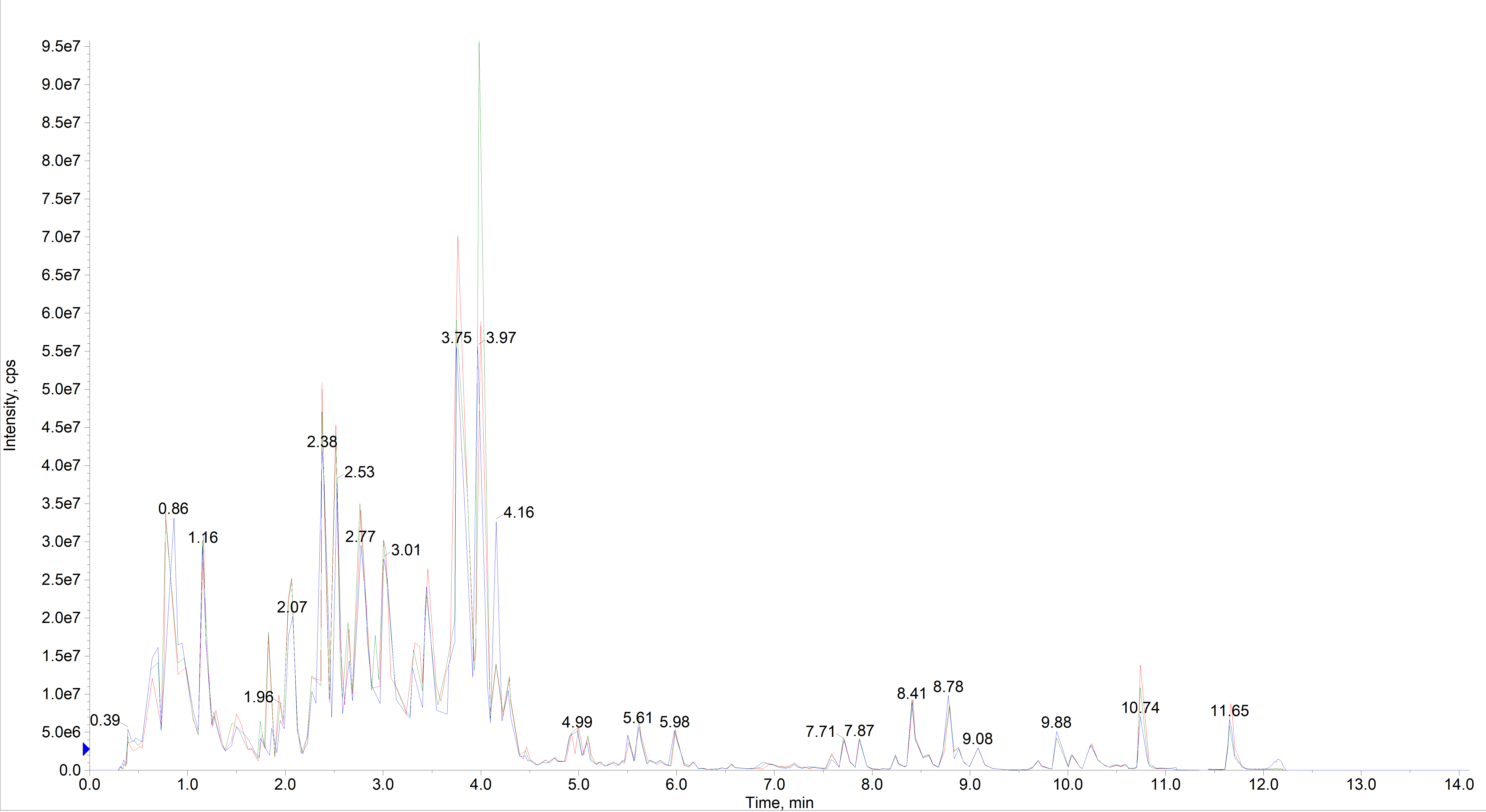

Supplement: Supplementary file 1 [file molecules-24-03323-s001.zip › supplementary files/Fig.S1a.jpg]

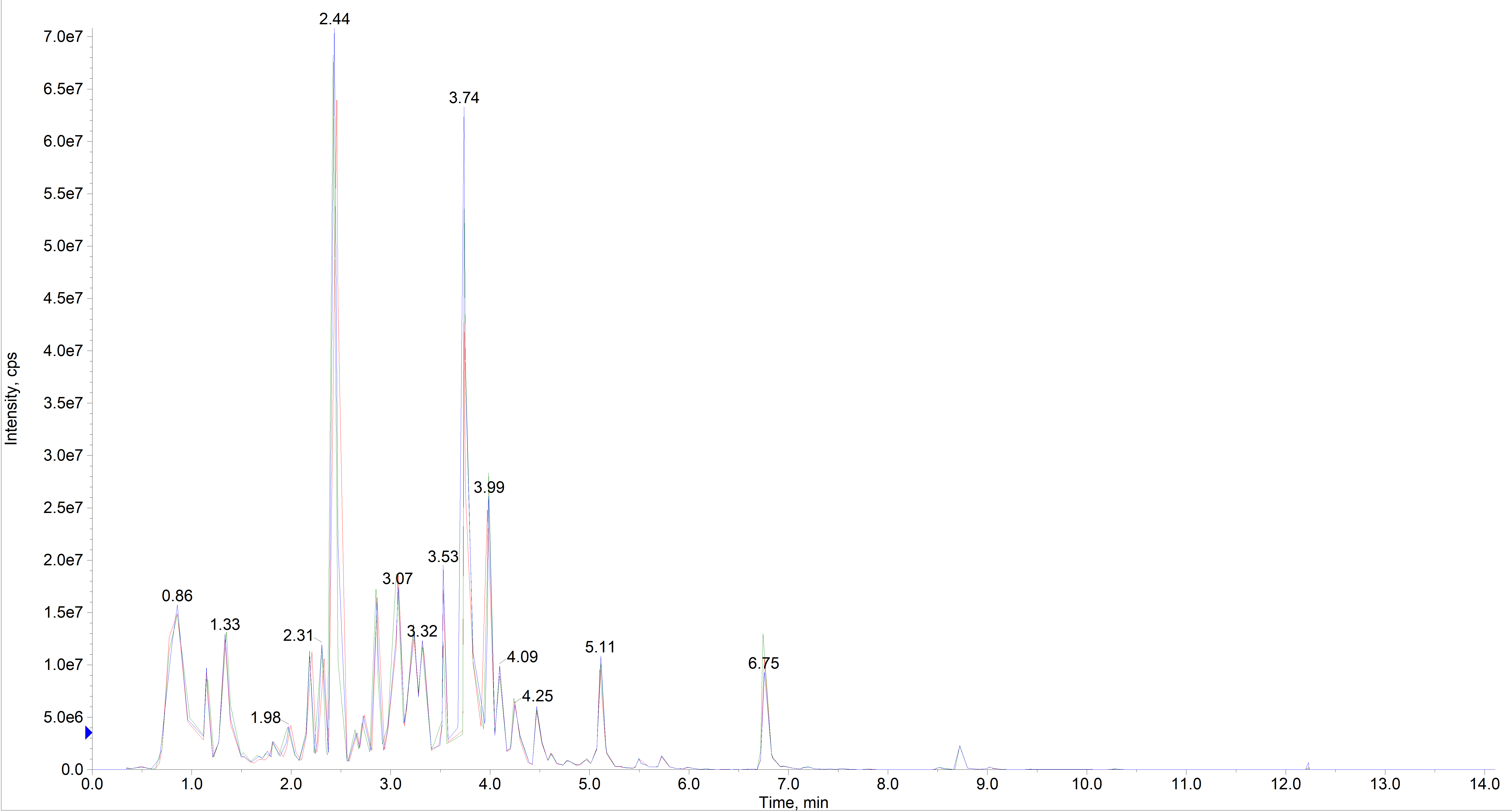

Supplement: Supplementary file 1 [file molecules-24-03323-s001.zip › supplementary files/Fig.S1b.jpg]
